# Small-Target Detection via Fusion of Visible and Infrared Image Features

**DOI:** 10.3390/s26175517

**Published:** 2026-08-31

**Authors:** Yu Dong, Chengxin Xie, Chaosheng Zhang, Qingsen Hu, Xue Han, Changzhu Xu

**Affiliations:** Department Engineering Equipment, Army Engineering University of PLA, Xuzhou 221004, China; dongyu@aeu.edu.cn (Y.D.); xiechengxin@aeu.edu.cn (C.X.); xuzhouhuqs@aeu.edu.cn (Q.H.); hanxue@aeu.edu.cn (X.H.); xcz@aeu.edu.cn (C.X.)

**Keywords:** visible and infrared fusion, small target detection, transformer, cross-attention, Focaler-SIoU

## Abstract

Visible–infrared small-target detection is challenged by weak single-modality representation, modality discrepancy, and the quadratic cost of dense cross-modal attention. We propose TFFB, a feature-level fusion detector that combines spatial feature compression (SFC), cross-attention modality enhancement (CME), and iterative cross-modal enhancement (ICME) to balance information exchange and computational efficiency. To further improve localization, we introduce Focaler-SIoU for small-box regression. On Anti-UAV300, TFFB improves the middle-fusion baseline from 76.2%/43.7% to 81.5%/48.6% in mAP@0.5/mAP@0.5:0.95, and TFFB with Focaler-SIoU reaches 83.6% and 50.2%, respectively. The results indicate that compact cross-modal interaction can strengthen visible–infrared UAV detection while keeping computational costs moderate.

## 1. Introduction

Object detection is a fundamental task in computer vision and requires both semantic category prediction and accurate bounding-box localization. For low-altitude small unmanned aerial vehicles (UAVs), these two subtasks are especially difficult because the target often occupies only a few pixels, exhibits weak texture and blurred edges, and is easily confused with backgrounds such as clouds, buildings, vegetation, and low-light night scenes. After repeated downsampling in deep detectors, the already weak target response can be further attenuated, which increases missed detections, false positives, and localization errors [1,2].

Visible and infrared sensors provide complementary information for this problem. Visible images contain fine texture, color, edge, and contour cues, but they are sensitive to illumination degradation, occlusion, and backlighting. Infrared images emphasize thermal radiation and are more stable in night or low-light conditions, but they usually contain fewer spatial details and may suffer from thermal diffusion. Therefore, the key research problem is not only how to combine two modalities but also how to produce stable and discriminative small-target representations while controlling the cost of cross-modal interaction.

The existing fusion strategies can be grouped into input-level, feature-level, and decision-level fusion. Input-level fusion is simple but may introduce raw noise and registration errors directly into the detector. Decision-level fusion is flexible in engineering deployment, but the two modalities interact only near the prediction stage and therefore cannot fully exploit shallow and middle-level complementary cues. Feature-level fusion is more suitable for small targets because it allows cross-modal information exchange during the progressive construction of semantic features. However, Transformer-based feature fusion can be computationally expensive when applied densely to high-resolution feature maps, and existing methods still require clearer separation between cross-modal interaction effectiveness and computational efficiency.

Motivated by these observations, we propose a visible–infrared feature-level fusion detector based on a dual Cross-Transformer Fusion Block (TFFB). The fundamental motivation is the insufficient and unstable representation of weak small targets in a single modality. The corresponding technical obstacles are modality discrepancy, insufficient cross-modal alignment, the high computational cost of dense attention, and localization instability for small bounding boxes. TFFB addresses these obstacles by combining spatial token compression, bidirectional cross-attention, and parameter-shared iterative refinement. Focaler-SIoU is further adopted to improve bounding-box regression for small targets.

The main contributions of this paper are summarized as follows:

(1) We propose a TFFB fusion module for visible–infrared small-target detection, enabling feature-level cross-modal interaction at multi-scale layers.
(2) We design three coordinated submodules—SFC, CME, and ICME—to reduce redundant spatial attention, explicitly exchange visible and infrared information, and deepen refinement through parameter sharing.
(3) We introduce Focaler-SIoU to enhance small-target bounding-box regression and clearly distinguish the contribution of TFFB alone from that of TFFB combined with Focaler-SIoU.
(4) We evaluate the proposed method on Anti-UAV300 and compare it with recent visible–infrared detectors, showing that the main gain is an improved accuracy–complexity trade-off within a fixed YOLOv11-RGBT framework.

## 2. Related Work

Small-target detection has been studied through multi-scale feature enhancement, contextual modeling, and improved regression losses. Two-stage detectors such as Faster R-CNN improve localization through region proposals [3], while one-stage detectors such as YOLO provide efficient end-to-end prediction [4]. FPN strengthens multi-scale semantic representation [5], and RetinaNet addresses dense foreground–background imbalance through Focal Loss [6]. Transformer-based detectors further introduce global modeling capabilities [7,8]. These approaches provide useful detection backbones, but single-modality images remain unreliable when UAV targets are tiny, low-contrast, or partially occluded.

Multispectral and RGB-T detection methods exploit complementary sensing cues. KAIST established an aligned visible–thermal pedestrian benchmark [9], while subsequent multispectral methods used joint detection–segmentation and multimodal feature learning to improve all-weather perception [10]. DroneVehicle further demonstrated the value of RGB–infrared complementarity for aerial scenes with scale variation and illumination changes [11]. Recent RGB-T methods such as GSMA-MS use grouped multi-receptive attention and multimodal supervision [12]. These studies show the benefits of dual-spectrum perception but also indicate that simple concatenation or late fusion is insufficient for weak small-target representation.

Attention and Transformer fusion have become important for cross-modal interaction. CBAM provides a lightweight channel-spatial attention reference [13]. CFT models long-range dependencies between RGB and infrared features using Transformer-based multimodal fusion [14], and RDCRNet improves RGB-T complementarity through cross-modal representation modeling [15]. Their advantages lie in richer cross-modal dependency modeling, but the computational cost and the distinction between feature interaction and parameter efficiency remain important limitations. In contrast, TFFB separates token compression, bidirectional cross-attention, and parameter-shared iterative refinement.

Recent Anti-UAV300 detectors, including DCAM-DETR, WFSD-Net, and CSFADet, report strong benchmark performance and show that detector-level design remains important beyond fusion alone. These results motivate our focus on a module-level fusion study within a YOLOv11-RGBT baseline.

## 3. Methodology

To address the issue of insufficient single-modal information representation in small target detection under complex backgrounds, we propose a dual-spectrum fusion target detection network based on the complementary characteristics of visible light and infrared images. Visible light images provide abundant texture, edge, and structural details, which enhance the discrimination capability between target contours and background features, and infrared images emphasize thermal radiation responses, demonstrating superior stability under low-light, nighttime, and complex illumination conditions. Integrating these two modalities into the target detection network strengthens feature expression capabilities for target regions during feature extraction while reducing interference from complex backgrounds. Therefore, this study employs a feature-level fusion strategy by introducing a TFFB module within the multi-scale feature layers of the detection network, enabling deep interaction between visible and infrared features to improve detection accuracy and robustness for small targets.

### 3.1. Overall Network Architecture

**Figure 1:**
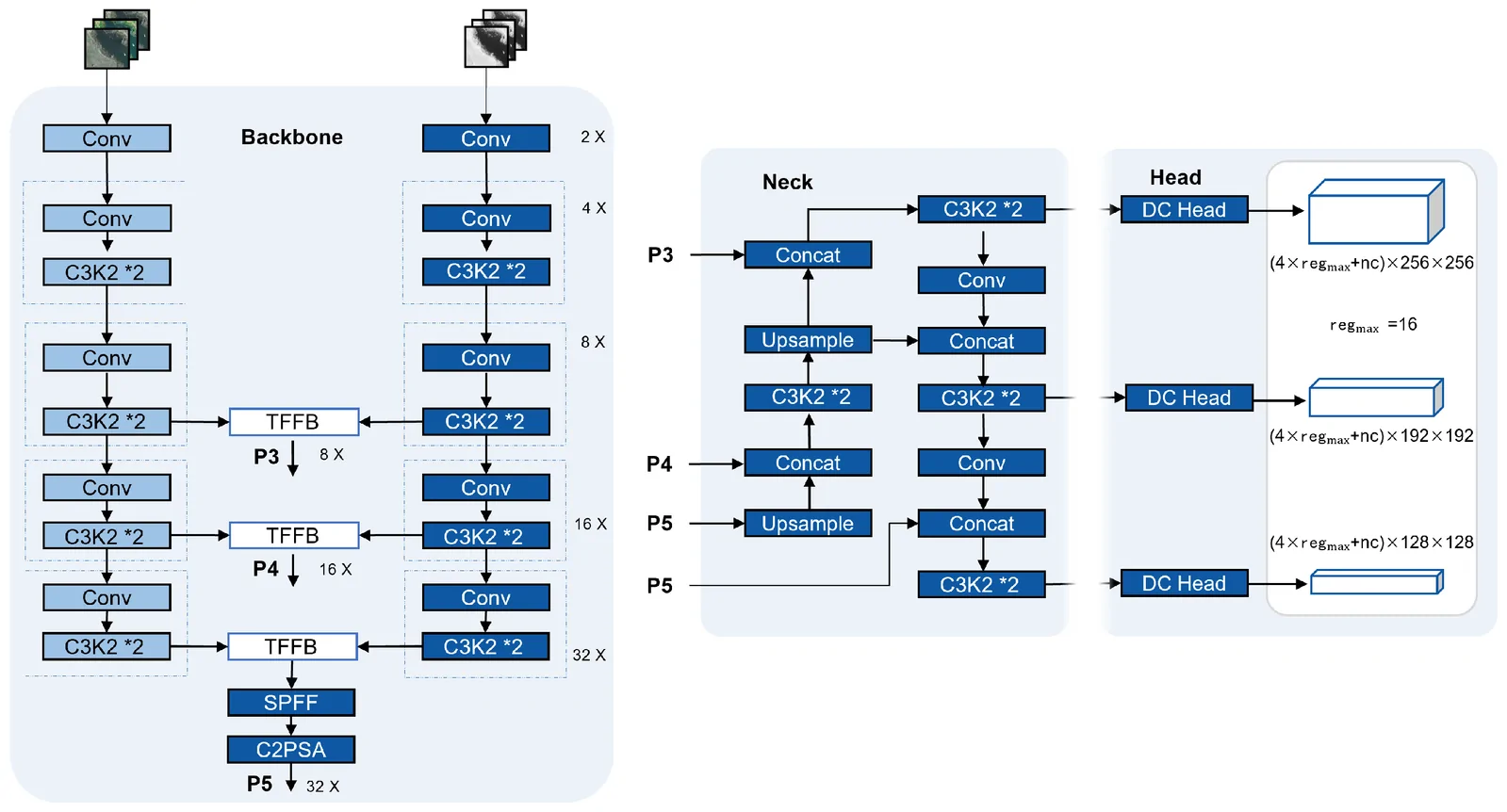
Detection framework with visible-infrared dual-branch feature fusion.

The proposed network consists of an input layer, a dual-branch backbone, the TFFB fusion module, a feature-aggregation neck, and decoupled detection heads. The overall network architecture is illustrated in Figure 1. Firstly, spatially registered visible and infrared images are separately fed into two structurally identical backbone branches to preserve the independent representation capabilities of both modalities. Subsequently, the TFFB module is introduced at three scales—shallow detail features (P3), mid-level fused features (P4), and deep semantic features (P5)—output by the backbone network, enabling cross-modal fusion in the mid-to-high-level semantic space. Finally, the fused multi-scale features are transmitted to the Neck for hierarchical aggregation, followed by classification and bounding-box regression performed through the decoupled detection head (DC Head).

**Figure 2:**
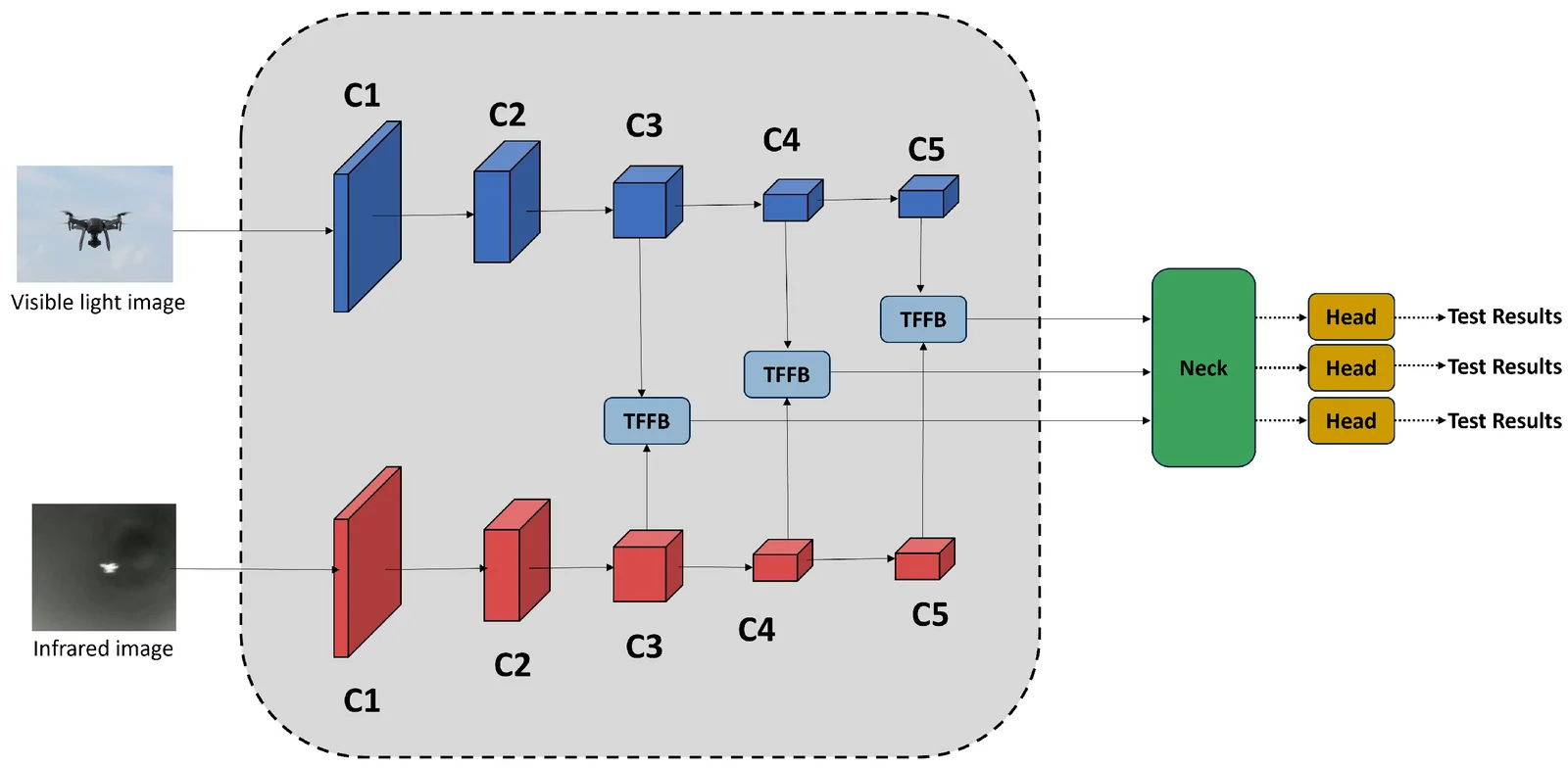
Architecture of the TFFB simplified module.

We also present a simplified representation of the overall network architecture, as illustrated in Figure 2. As shown in Figure 2, the proposed method adopts a dual-branch backbone network structure specifically designed for joint feature modeling of RGB-infrared image pairs. This architecture preserves the independent feature representation capabilities of both modalities while introducing cross-modal feature interaction and fusion mechanisms through the TFFB module. Consequently, it fully exploits the complementarity between visible light information and infrared information, providing more comprehensive and robust feature representations for subsequent multi-scale feature aggregation and object detection.

### 3.2. Feature Extraction

Unlike directly concatenating images at the input level, we opt for feature-level fusion. This design not only avoids the sensitivity of early fusion to registration errors and original noise but also overcomes the issue of too-late modality interaction in decision-level fusion, thereby being more conducive to the joint representation of weak target regions. From an overall workflow perspective, the proposed method primarily consists of three stages: unimodal feature extraction, bimodal feature fusion, and detection neck with detection head inference. Specifically, during the unimodal feature extraction stage, features are first extracted separately from RGB images and infrared images, which can be mathematically formulated as:

(1) $$F_{R}^{i}=\Psi_{\mathrm{backbone}}\left(I_{R};\theta_{R}\right),\;F_{T}^{i}=\Psi_{\mathrm{backbone}}\left(I_{T};\theta_{T}\right)$$

where $F_{R}^{i},F_{T}^{i}\in R^{W\times H\times C}$ denote the feature maps from the RGB branch and the infrared branch at the *i*-th layer i=3,4,5, respectively, and *W*, *H*, and *C* represent the width, height, and number of channels of the feature maps, respectively. $I_{R},I_{T}\in R^{W\times H\times C}$ denote the input images. Subscript backbone represents the feature extraction functions for the RGB and infrared branches, with parameters $\theta_{R}$ and $\theta_{T}$, respectively.

The above process obtains multi-scale feature representations of the RGB image and the infrared image, respectively. However, the two modalities differ in their imaging mechanisms and feature responses, making it difficult for separate modeling to fully exploit their complementary information. To further enhance the feature representation capability for weak and small targets, we introduce the TFFB module at the feature level to perform cross-modal interaction and fusion between RGB and infrared features.

### 3.3. Dual Cross-Transformer Fusion Block

**Figure 3:**
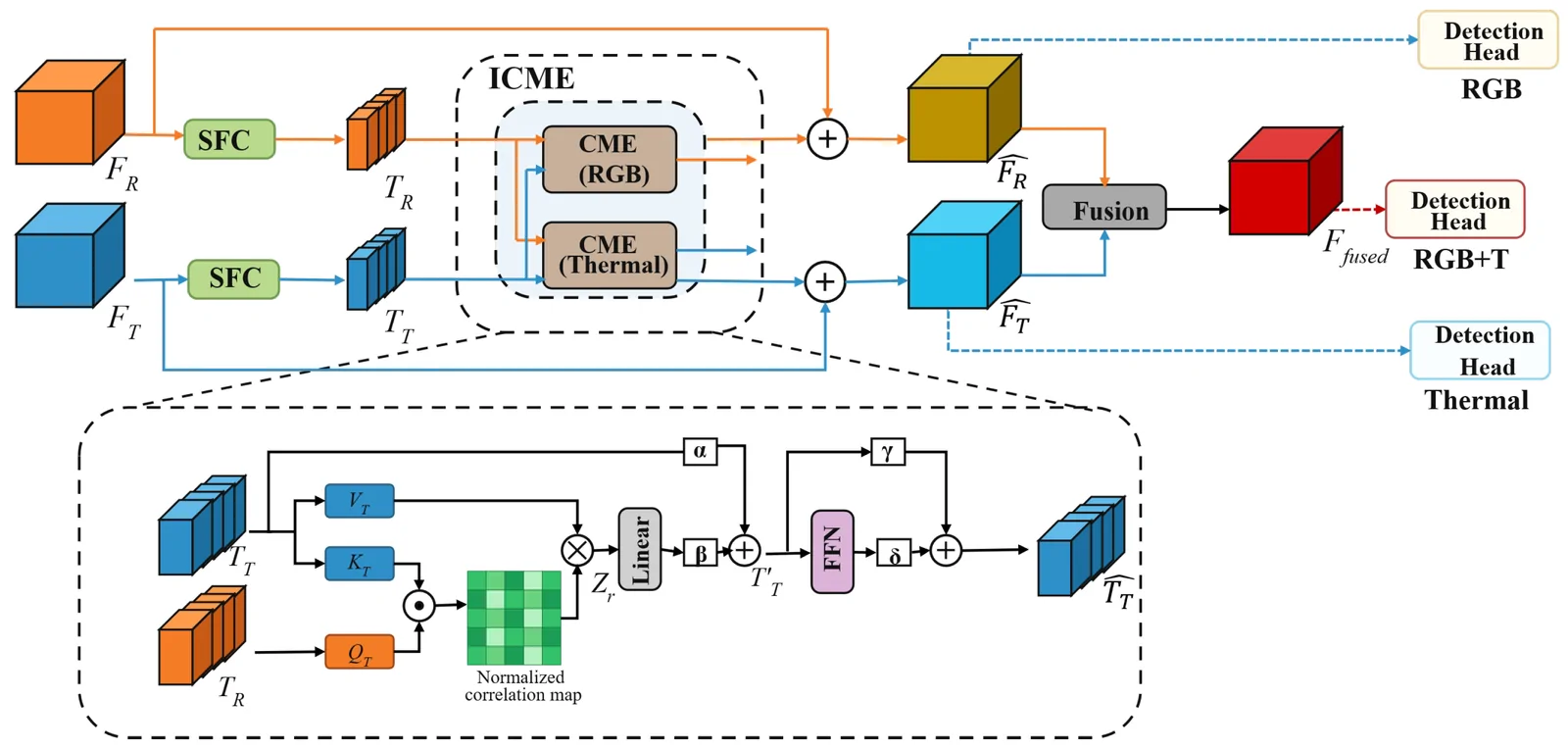
Schematic diagram of the TFFB fusion module structure.

TFFB serves as the core module of the proposed method, comprising three components: Spatial Feature Compression (SFC), Cross-Attention Modal Enhancement (CME), and Iterative Cross-Modal Enhancement (ICME). The SFC module reduces spatial redundancy in input feature maps, while CME establishes explicit global correlations between the two modalities. ICME progressively refines fused features through multi-round interactions with parameter sharing. The structural configuration of the TFFB fusion module is illustrated in Figure 3.

#### 3.3.1. Spatial Feature Compression Module (SFC)

In a Transformer, the computational complexity of attention is quadratic with respect to the number of tokens. When the resolution of the feature map is high, directly performing cross-modal attention leads to large memory consumption and computational overhead. Therefore, we introduce an SFC module before CME, which compresses the feature map from the spatial size of H×W to H/s×W/s, thereby reducing the number of tokens from HW to $HW/s^{2}$, where *s* denotes the spatial downsampling stride of the SFC compression module. This module can be implemented in two ways: convolution-based compression or mixed pooling. Convolution-based compression selects discriminative channels through spatial-to-channel rearrangement and a 1×1 convolution, while mixed pooling balances global background information and local salient responses through learnable weighting of average pooling and max pooling. For the reproducibility specification used in the revised implementation, the mixed-pooling form is fixed with a spatial stride of s=2, corresponding to a fourfold reduction in spatial token count. The learnable pooling coefficient is initialized at λ=0.5 and constrained to [0,1] during optimization.

Considering the differences between visible-light and infrared features in representation information and response intensity, a single pooling method may not simultaneously preserve background information and target details. Therefore, inspired by mixed pooling, we propose a spatial contraction method that adaptively fuses average pooling and max pooling. Its mathematical formulation is shown as follows:

(2) $$\begin{array}{cc} F_{o} & =\lambda \cdot F_{a}+\left(1-\lambda \right)\cdot F_{m}. \end{array}$$

Here the feature maps compressed by average pooling and max pooling are denoted as $F_{a}$ and $F_{m}$, respectively, and are computed as follows: $F_{a}=AvgPooling\left(F,S\right)$ and $F_{m}=MaxPooling\left(F,S\right)$, where *F* denotes the input feature map, and *S* denotes the scaling factor of the feature map. And λ is a learnable weight parameter with a value range from 0 to 1, which is used to adaptively adjust the contribution ratio of the two pooling results in the final output. By introducing the learnable parameter, the model can automatically select a more suitable feature compression strategy according to the training data, thereby improving the robustness of the compressed features.

#### 3.3.2. Cross-Attention Modality Enhancement Module (CME)

The CME module actively mines complementary information from the auxiliary modality through a cross-modal attention mechanism, thereby achieving bidirectional enhancement of visible-light and infrared features. Taking visible-light feature enhancement as an example, the model uses the compressed visible-light token sequence $X_{v}$ as the query and the infrared token sequence $X_{r}$ as the key and value. Through cross-modal attention computation, guidance information from the infrared modality to the visible-light modality is obtained. This process can utilize the thermal response features in infrared images to supplement the information representation of visible-light images under insufficient illumination or missing texture conditions, thereby enhancing the texture details and target response capability of the visible-light branch. The calculation process is as follows:

(3) $$\begin{array}{cc} Q & =X_{v}W_{Q},\;K=X_{r}W_{K},\;V=X_{r}W_{V},\\ A & =\mathrm{Softmax}\left(\frac{QK^{T}}{\sqrt{d_{k}}}\right),\;Z=AV,\\ Y & =X_{v}+\gamma \cdot \mathrm{Proj}\left(Z\right) \end{array}$$

where $X_{v}$ and $X_{r}$ denote the compressed visible-light and infrared token sequences, respectively. $W_{Q}$, $W_{K}$, and $W_{V}$ are linear projection matrices, $d_{k}$ denotes the dimensionality of the key vectors in a single attention head, γ is a learnable residual scaling coefficient, and Proj(·) denotes the output projection operation. CME uses eight parallel cross-attention heads, consistent with the original module design; hence, $d_{k}=C/8$ when the projected embedding dimension is *C*. The residual scaling coefficient is initialized to γ=1 and is optimized jointly with the remaining network parameters. Through the above mechanism, the thermal response information in the infrared modality can effectively supplement the texture details of the visible-light modality, thereby improving the representation quality of visible-light features.

#### 3.3.3. Iterative Cross-Modal Enhancement Module (ICME)

**Figure 4:**
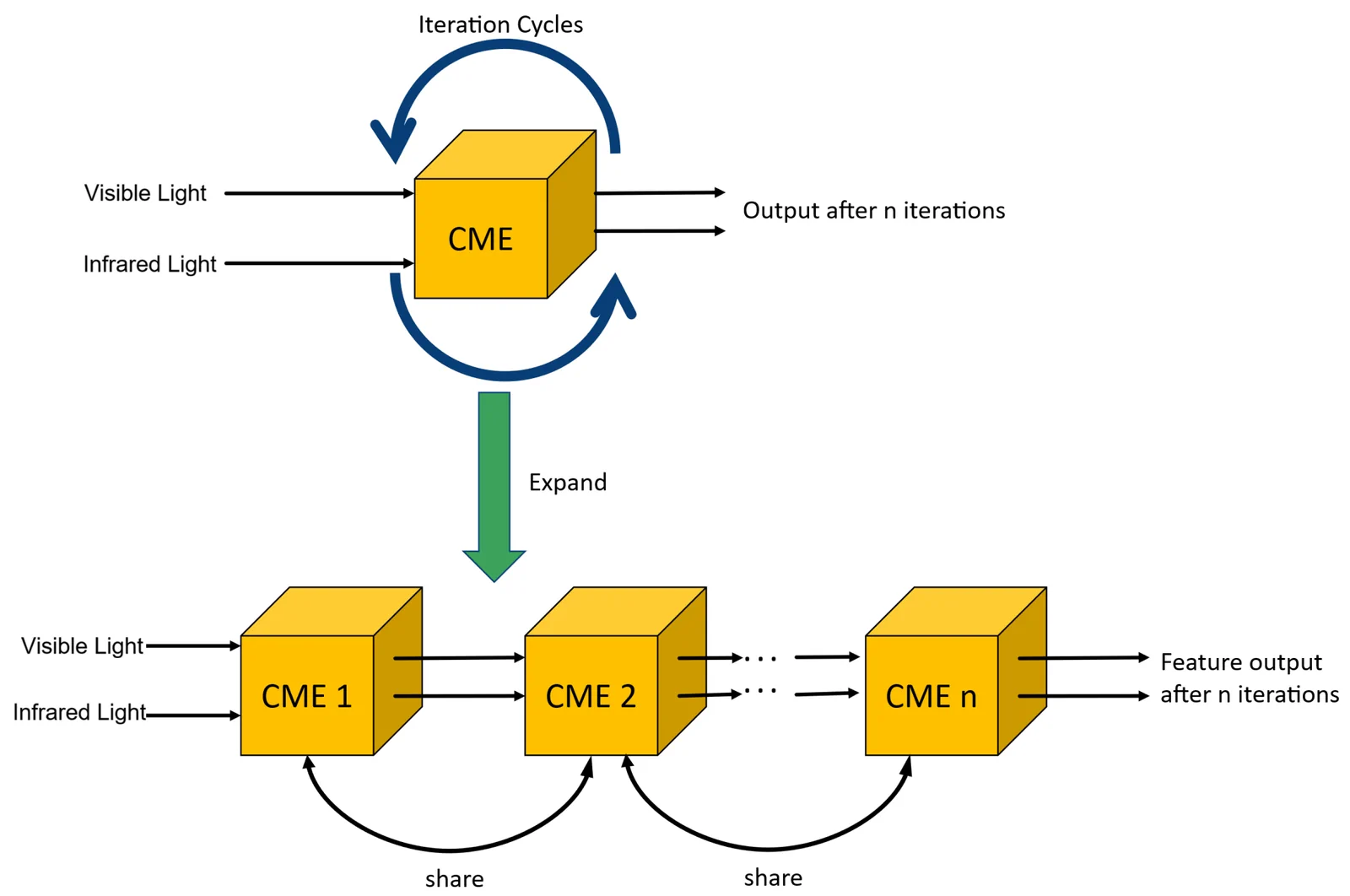
The ICME parameter-sharing iterative enhancement mechanism.

As shown in Figure 4, this mechanism repeatedly uses the same set of CME parameters across multiple iterative steps to perform multiple rounds of cross-modal enhancement and refinement on the features. Unlike traditional stacking methods, ICME does not simply increase the number of network layers. Instead, it repeatedly revisits the feature representations, enabling the network to gradually deepen the cross-modal feature interaction without introducing additional parameters. Theoretically, this design is equivalent to performing multiple nonlinear transformations on feature representations within a constrained parameter space, thereby achieving more sufficient feature optimization.

This strategy does not simply increase the network depth. Instead, it repeatedly uses the same set of cross-modal feature enhancement modules through multiple iterations to progressively refine the features. In this way, the network achieves deeper feature interaction without introducing additional parameters, thereby obtaining a better balance between model complexity and performance improvement.

Assume that the initial input feature sequences of the ICME module undergo *n* iterations. The feature update process can be expressed as:

(4) $$\{{\hat{T}}_{R}^{n},{\hat{T}}_{T}^{n}\}=F_{ICME}(\{T_{R},T_{T}\},n)=\overset{F_{CME}(\cdots F_{CME}(\{T_{R},T_{T}\}))}{\underset{n}{︸}}$$

where $\left\{{\hat{T}}_{R}^{n},{\hat{T}}_{T}^{n}\right\}$ denotes the output sequences obtained after *n* iterations, and $F_{ICME}\left(\cdot \right)$ denotes the iterative cross-modal refinement mapping. In this work, ICME applies bidirectional CME-based enhancement to the RGB and thermal branches. The output of each iteration is used as the input to the next iteration, while the CME parameters are shared across all iterations. The ICME iteration number is fixed to n=2. Thus, after the first CME interaction, one additional parameter-shared refinement round is performed. This setting provides a practical accuracy–complexity trade-off: relative to the single-CME configuration in Table 7, the complete TFFB increases FLOPs from 19.4G to 20.5G while improving mAP@0.5:0.95 from 47.1% to 48.6%. We, therefore, use two iterations in all controlled experiments, without claiming that n=2 is universally optimal for other datasets or detector scales. In addition, the ICME output sequences ${\hat{T}}_{R}^{n}$ and ${\hat{T}}_{T}^{n}$ are first converted into feature maps and then resized to the original resolution through bilinear interpolation to ensure feature consistency.

#### 3.3.4. Methodological Distinction from CFT and RDCRNet

The proposed fusion module differs from existing cross-modal attention approaches in the way it balances interaction depth and computational cost. CFT performs Transformer fusion over multimodal feature tokens and is effective for modeling long-range dependencies, but dense attention over high-resolution feature maps increases computational cost. TFFB first uses SFC to reduce the spatial token count from HW to $HW/s^{2}$, so the dominant attention term decreases from approximately $O({\left(HW\right)}^{2})$ to $O({\left(HW/s^{2}\right)}^{2})$. CME then performs explicit bidirectional cross-attention on the compressed token representations rather than dense multimodal self-attention at the original resolution. Compared with RDCRNet, which emphasizes cross-modal representation learning, TFFB explicitly separates computational reduction (SFC), single-round interaction (CME), and repeated refinement (ICME). The ICME rounds reuse the same CME parameters, which increases the depth of cross-modal refinement without allocating an independent Transformer parameter set to every round. Therefore, the empirical comparison with CFT in the common YOLOv11-RGBT framework should be interpreted as evidence of an accuracy–complexity trade-off, not as a universal runtime guarantee.

### 3.4. Focaler-SIoU Bounding-Box Regression Loss

Low-altitude, slow-moving, and small targets usually occupy only a small number of pixels in an image. A slight offset between the predicted bounding box and the ground-truth box can significantly affect the IoU metric. In addition, thermal diffusion and blurred visible-light edges further aggravate the instability of bounding-box regression. To improve the model’s sensitivity to difficult samples and localization errors of small targets, we introduce the Focaler-SIoU loss function into the detection head. This loss combines the angular, distance, and shape geometric constraints of SIoU [16] with the hard-sample focusing capability of the Focaler mechanism [17], enabling the model to pay more attention to medium- and low-IoU samples during training. The Focaler-SIoU loss function is formulated as follows:

(5) $$L_{\mathrm{Focaler}\text{-}\mathrm{SIoU}}=L_{\mathrm{SIoU}}+\mathrm{IoU}-{\mathrm{IoU}}^{\mathrm{focaler}}$$

where $L_{\mathrm{SIoU}}$ denotes the SIoU loss, which incorporates overlap, angle-aware center displacement, distance, and shape information to constrain bounding-box regression. In Equation (5), IoU denotes the conventional intersection-over-union value, while ${\mathrm{IoU}}^{\mathrm{focaler}}$ is the remapped IoU defined by the Focaler-IoU mechanism. The combination preserves the geometric constraints of SIoU while adjusting the contribution of regression samples according to their IoU range.

(1) SIoU Loss Function

SIoU is an IoU-based bounding-box regression loss that explicitly considers the direction of the center-point displacement between the predicted and ground-truth boxes. In addition to the IoU overlap term, SIoU introduces angle, distance, and shape costs, providing richer geometric guidance for localization than conventional IoU alone. This property is useful for small-target detection, where even a small center-point offset can cause a substantial decrease in IoU. The overall loss function is defined as:

(6) $$L_{\mathrm{SIoU}}=1-\mathrm{IoU}+\frac{\Delta +\Omega }{2}$$

where Δ denotes the distance cost term, and Ω denotes the shape cost term. The SIoU loss is jointly composed of the IoU cost, angle cost, distance cost, and shape cost.

(2) Focaler-IoU Loss Function

Focaler-IoU is an IoU-based regression loss that considers the distribution of easy and difficult bounding-box regression samples. Through linear interval mapping and clipping of the IoU value, it changes the relative emphasis assigned to samples in different IoU ranges. In this work, the mapping is used to increase attention to regression samples that remain informative for improving small-target localization. Its calculation process can be expressed as:

(7) $$L_{\mathrm{Focaler}\text{-}\mathrm{IoU}}=1-{\mathrm{IoU}}^{\mathrm{focaler}}$$

(8) $${\mathrm{IoU}}^{\mathrm{focaler}}=\{\begin{array}{cc} 0, & \mathrm{IoU}\leq d,\\ \frac{\mathrm{IoU}-d}{u-d}, & d<\mathrm{IoU}<u,\\ 1, & \mathrm{IoU}\geq u. \end{array}$$

where [d,u]∈[0,1] and 0≤d<u≤1. In the experiments, we set d=0.00 and u=0.95, following the default setting of the original Focaler-IoU implementation. The lower threshold *d* defines the start of the active linear-mapping interval, whereas *u* defines the saturation point for already well-aligned predictions. When IoU≤d, the mapped value ${\mathrm{IoU}}^{\mathrm{focaler}}$ is clipped to 0 and the corresponding Focaler-IoU loss $L_{\mathrm{Focaler}\text{-}\mathrm{IoU}}$ equals 1. When d<IoU<u, the mapped value increases linearly and emphasizes medium-quality samples, which are often more informative for improving small-target localization. When IoU≥u, the mapped value is clipped to 1 and the Focaler-IoU term equals 0, rather than 1. The complete Focaler-SIoU objective in Equation (5) still contains the SIoU and explicit IoU terms, so the whole regression objective is not removed outside the active Focaler interval; only the Focaler-IoU focusing term is clipped.

## 4. Experiments and Result Analysis

### 4.1. Dataset and Evaluation Metrics

The experiments use the anti-UAV300 visible–infrared low-altitude UAV dataset [1]. Following the processed source package, frames are sampled from the original video sequences every 20 frames. The resulting paired-image split is summarized in Table 1. The dataset includes complex backgrounds, diverse illumination conditions, and UAV targets at different scales, making it suitable for evaluating visible–infrared small-target detection.

#### Training Protocol and Reproducibility

Mosaic and MixUp augmentations were used to improve sample diversity. For reproducibility, the revised implementation fixes the training protocol as shown in Table 2: paired RGB and infrared inputs are resized to 640×640 pixels; both backbone branches are initialized from COCO-pretrained YOLO11 weights; training is performed for 100 epochs with a batch size of 16 using SGD. The initial learning rate is 0.01 and follows cosine annealing to $1\times 10^{-4}$, with momentum 0.937, weight decay $5\times 10^{-4}$, and a 3-epoch warm-up. Automatic mixed precision is enabled, the dataloader uses eight workers, and the random seed is fixed to 0. For all of the controlled comparisons below, all models use the same YOLOv11-RGBT backbone [18], processed split, augmentation pipeline, detection head, and training protocol; only the fusion strategy or fusion module is changed. The experiments are conducted on an AMD Ryzen 7 7800X3D CPU with 32 GB RAM and an NVIDIA GeForce RTX 5070 GPU with 12 GB VRAM, running Windows 11, PyTorch, CUDA 12.8, cuDNN 9.19.1, and Python 3.11. The evaluation metrics are Precision, Recall, F1, mAP@0.5, mAP@0.5:0.95, and FLOPs.

### 4.2. Controlled Comparison of Fusion Stages

To further investigate the influence of fusion stages on visible–infrared small target detection, we refer to the multi-stage cross-modal fusion framework reported by Zhu et al. [19] and construct three representative fusion strategies based on YOLOv11 [18]: early fusion, middle fusion, and late fusion. In the early-fusion strategy, visible and infrared images are integrated at the input level, enabling the network to learn complementary low-level features from the two modalities. In the middle-fusion strategy, modal features are first extracted separately and then fused in the intermediate feature-extraction stage, allowing the model to combine semantic and spatial information more effectively. In the late-fusion strategy, the detection results or high-level semantic features from the two modalities are fused near the prediction stage.

Table 3 is a controlled comparison: the dataset split, backbone, detector head, augmentation, and available training protocol were held fixed, while the fusion stage was changed. It evaluates the effect of fusion position within the common YOLOv11-RGBT experimental framework; it is not a comparison with results reported by other papers.

As shown in Table 3, after combining middle fusion with TFFB, all metrics outperform the baselines of early fusion, middle fusion, and late fusion. Specifically, mAP@0.5 improves by 5.3 percentage points compared with ordinary middle fusion, indicating that introducing cross-modal attention at the feature level can effectively enhance the semantic representation and background suppression capability for slow-moving and small targets.

### 4.3. Controlled Comparison with Fusion Modules

For the middle-fusion strategy, the proposed TFFB is compared with CFT [14], CAS [20], MCF [21], and EFM [22]. This is a controlled fusion-module comparison: all methods are integrated into the YOLOv11-RGBT framework and evaluated on the same processed Anti-UAV300 split, with the fusion module as the intended experimental variable. The results are shown in Table 4.

As shown in Table 4, TFFB achieves the highest detection accuracy while maintaining a moderate computational cost. Compared with CFT, the FLOPs of TFFB decrease from 28.3G to 20.5G, while mAP@0.5:0.95 improves by 1.5 percentage points. This indicates that the spatial compression and parameter-sharing iterative mechanism can reduce redundant computation while maintaining strong cross-modal modeling capability.

### 4.4. Cross-Paper Comparison with Recent Anti-UAV300 Methods

Table 5 is a cross-paper comparison. It summarizes published Anti-UAV300 results for recent visible–infrared UAV detectors and the proposed method. Because the architectures, training protocols, input resolutions, data processing, and runtime environments may differ across papers, these values contextualize the benchmark landscape and must not be interpreted as a controlled ablation or a direct measure of the isolated effect of TFFB.

The cross-paper comparison shows that the proposed complete detector is not the current state of the art on Anti-UAV300. The main empirical contribution of this work is narrower and is supported by the controlled experiments: under the fixed YOLOv11-RGBT framework, replacing the baseline fusion module with TFFB improves mAP@0.5:0.95 from 43.7% to 48.6% while requiring 20.5G FLOPs, and it outperforms CFT in the same fusion-module setting.

### 4.5. Effect of Loss Function Improvement

Table 6 shows that Focaler-SIoU provides stable improvements for various fusion strategies, and the improvement is most significant when it is combined with TFFB. The mAP@0.5 of TFFB + Focaler-SIoU reaches 83.6%, and the mAP@0.5:0.95 reaches 50.2%. This indicates that the loss function can further transform the target response advantages in the fused features into more accurate bounding-box localization.

### 4.6. Ablation Study

The proposed TFFB consists of three components: SFC, CME, and ICME. SFC compresses redundant spatial information and reduces computational cost; CME establishes cross-modal interaction between visible-light and infrared features; and ICME further refines the fused features through a parameter-sharing iterative mechanism based on CME. ICME is not a parallel module independent of CME, but a multi-round enhancement strategy built upon CME. This section, therefore, uses middle fusion as the baseline and sequentially introduces SFC, CME, and the complete TFFB to analyze the contribution of each component. All experiments use the same Anti-UAV300 split, fixed training protocol, and evaluation metrics. This structural ablation does not introduce Focaler-SIoU, thereby separating structural improvement from loss-function improvement. The complete TFFB uses two ICME iterations (n=2) in all controlled experiments. The comparison between the single-CME row and the complete TFFB row quantifies the gain obtained by adding the second parameter-shared refinement round.

As shown in Table 7, SFC, CME, and ICME all have positive effects on performance improvement. SFC improves detection accuracy while slightly reducing FLOPs, indicating that compressing redundant spatial information is beneficial for subsequent fusion. CME brings the main performance gain, demonstrating that bidirectional cross-attention is the key to modeling cross-modal complementary information. ICME further improves mAP with only a small increase in computational cost, indicating that parameter-sharing iterative enhancement can further refine target-region features.

## 5. Discussion

The experimental results indicate that the advantage of TFFB mainly comes from three aspects. First, feature-level fusion avoids directly amplifying raw sensor noise while also avoiding the insufficient interaction of decision-level fusion. Second, SFC reduces the token count before attention, allowing CME to establish bidirectional correspondences between visible textures and infrared thermal responses with lower computational overhead than dense high-resolution attention. Third, ICME repeats cross-modal enhancement with shared parameters, improving interaction depth without introducing a separate Transformer parameter set at every refinement round.

The present study is validated on Anti-UAV300, so its conclusions are limited to a single benchmark. The revised manuscript now specifies a fixed training and implementation protocol, including input resolution, optimizer, learning-rate schedule, batch size, training epochs, SFC stride, CME heads, and ICME iterations. Broader claims about generalization and deployment efficiency will require additional RGB–T datasets, runtime profiling, and memory analysis. Future work will, therefore, focus on registration-aware fusion, temporal modeling, cross-dataset evaluation, and deployment-oriented benchmarking.

Future work should include registration-aware or deformable cross-modal attention to handle imperfect RGB–infrared alignment, temporal modeling for video UAV detection, cross-dataset evaluation on additional RGB-T benchmarks, qualitative detection or feature-map visualization under night, low-light, cloud, and cluttered backgrounds, and deployment-oriented measurements including parameter count, FPS/latency, and peak memory under a fixed runtime protocol.

## 6. Conclusions

This paper proposes a visible–infrared small-target detection method based on TFFB and Focaler-SIoU. TFFB combines SFC, CME, and ICME to integrate spatial token compression, bidirectional cross-modal attention, and parameter-shared iterative refinement. In the YOLOv11-RGBT experiments, TFFB alone improves the middle-fusion baseline from 76.2%/43.7% to 81.5%/48.6% in mAP@0.5/mAP@0.5:0.95. Adding Focaler-SIoU further improves the final detector to 83.6% and 50.2%, respectively. These results support the effectiveness of the proposed fusion module and regression loss within the evaluated Anti-UAV300 setting. Broader generalization and practical runtime efficiency will be examined on additional RGB–T benchmarks and under unified deployment-oriented measurement protocols.

## Figures and Tables

**Table 1:** Processed anti-UAV300 split used in this work.

Subset	Number of Paired Images
Training	5104
Validation	2209
Testing	2203

**Table 2:** Training and implementation parameters for the controlled experiments.

Parameter	Setting
Input resolution	640×640 for both RGB and infrared inputs
Initialization	COCO-pretrained YOLO11 weights for both backbone branches
Optimizer	SGD
Initial/final learning rate	0.01/$1\times 10^{-4}$
Learning-rate schedule	Cosine annealing with 3-epoch warm-up
Momentum/weight decay	0.937/$5\times 10^{-4}$
Batch size/training epochs	16/100
Random seed/AMP/workers	0/enabled/8
Data augmentation	Mosaic and MixUp
SFC spatial stride	s=2
CME attention heads	8 ($d_{k}=C/8$)
ICME iterations	n=2, with CME parameters shared across rounds
Focaler-IoU interval	d=0.00, u=0.95

**Table 3:** Detection performance comparison of different fusion-stage strategies. The best result in each column is in bold.

Fusion Strategy	Precision	Recall	mAP@0.5	mAP@0.5:0.95
Early Fusion	81.2%	68.5%	74.6%	42.1%
Middle Fusion	82.6%	70.1%	76.2%	43.7%
Late Fusion	83.4%	71.8%	77.4%	45.1%
Middle Fusion + TFFB	**86.8%**	**76.5%**	**81.5%**	**48.6%**

**Table 4:** Performance and computational cost comparison between TFFB and mainstream feature fusion modules. The best result in each column is in bold.

Method	Precision	Recall	F1	mAP@0.5	mAP@0.5:0.95	FLOPs/G
TFFB	**86.8%**	**76.5%**	**81.3%**	**81.5%**	**48.6%**	**20.5**
CFT	85.6%	74.8%	79.8%	80.2%	47.1%	28.3
CAS	84.5%	73.2%	78.5%	78.5%	45.8%	18.7
MCF	83.6%	72.1%	77.4%	77.3%	44.6%	15.2
EFM	83.0%	71.2%	76.6%	76.8%	44.0%	16.4

**Table 5:** Cross-paper reported Anti-UAV300 results of recent visible–infrared UAV detection methods.

Method	mAP@0.5	mAP@0.5:0.95	Notes
DCAM-DETR [23]	94.7%	78.3%	Reported 42 FPS
WFSD-Net [24]	96.0%	58.3%	Wavelet-guided frequency–spatial decoupling
CSFADet [25]	91.4%	58.7%	Cross-spectral alignment and adaptive refinement
TFFB + Focaler-SIoU (ours)	83.6%	50.2%	YOLOv11-RGBT setting

**Table 6:** Performance changes of different fusion strategies after introducing Focaler-SIoU. The best result in each column is in bold.

Fusion Strategy	Precision	Recall	F1	mAP@0.5	mAP@0.5:0.95
Early Fusion	81.2%	68.5%	74.3%	74.6%	42.1%
Early Fusion + Focaler-SIoU	82.5%	70.2%	75.9%	76.4%	43.5%
Middle Fusion	82.6%	70.1%	75.8%	76.2%	43.7%
Middle Fusion + Focaler-SIoU	83.9%	71.8%	77.4%	78.1%	44.9%
Late Fusion	83.4%	71.8%	77.2%	77.4%	45.1%
Late Fusion + Focaler-SIoU	84.6%	73.5%	78.7%	79.3%	46.6%
TFFB	86.8%	76.5%	81.3%	81.5%	48.6%
TFFB + Focaler-SIoU	**88.2%**	**78.1%**	**82.8%**	**83.6%**	**50.2%**

**Table 7:** Ablation results of TFFB component modules. The best result in each column is in bold.

Model Setting	SFC	CME	ICME	Precision	Recall	F1	mAP@0.5	mAP@0.5:0.95	FLOPs/G
Middle Fusion	×	×	×	82.6%	70.1%	75.8%	76.2%	43.7%	18.6
Middle Fusion + SFC	✓	×	×	83.3%	71.0%	76.7%	77.0%	44.2%	18.4
Middle Fusion + SFC + CME	✓	✓	×	85.4%	74.6%	79.6%	80.0%	47.1%	19.4
TFFB	✓	✓	✓	**86.8%**	**76.5%**	**81.3%**	**81.5%**	**48.6%**	**20.5**

## Data Availability

The Anti-UAV300 dataset analyzed in this study is publicly available, with the corresponding source cited in the References section. The processed image pairs used in the experiments were derived from the public dataset according to the frame-sampling and split protocol described in Section 4.1.
